# Association of Early Life Exposure to Antibiotics With Risk of Atopic Dermatitis in Sweden

**DOI:** 10.1001/jamanetworkopen.2021.5245

**Published:** 2021-04-29

**Authors:** Mwenya Mubanga, Cecilia Lundholm, Brian M. D’Onofrio, Marlene Stratmann, Anna Hedman, Catarina Almqvist

**Affiliations:** 1 Department of Medical Epidemiology and Biostatistics, Karolinska Institutet, Stockholm, Sweden; 2 Department of Psychological & Brain Sciences, Indiana University–Bloomington; 3 Pediatric Allergy and Pulmonology Unit at Astrid Lindgren Children’s Hospital, Karolinska University Hospital, Stockholm, Sweden

## Abstract

**Importance:**

Atopic dermatitis is associated with substantial morbidity in childhood. Further understanding of the underlying factors contributing to its onset is needed.

**Objective:**

To assess the association of exposure to antibiotics in the prenatal period and early childhood with risk of atopic dermatitis in a nationwide population in Sweden.

**Design, Setting, and Participants:**

This Swedish nationwide, register-based, prospective cohort study used data on mother-child pairs from the Swedish Medical Birth Register linked to other national registers for information on health, socioeconomic, and demographic data. Participants were followed up until an atopic dermatitis outcome, emigration, death, or the end of the study on December 31, 2015. Data for all singleton children and discordant siblings born between March 1, 2006, and December 31, 2010, were included. Data were analyzed from June 1, 2020, to October 31, 2020.

**Exposures:**

Maternal exposure to systemic antibiotics during pregnancy as well as the child’s exposure to systemic antibiotics during the first year of life, as defined by a dispensed prescription in the Swedish Prescribed Drug Register.

**Main Outcomes and Measures:**

Time-to-event analyses were used to estimate the risk of outcome using attained age as a time scale. Atopic dermatitis was defined based on diagnoses in the National Patient Register and medication listed in the Swedish Prescribed Drug Register. Sibling-control analysis was performed to account for shared familial factors.

**Results:**

Among the 722 767 singleton children, the mean (SD) age was 5.8 (2.4) years and 351 589 (48.6%) were female. During the follow-up period, 153 407 children (21.2%) were exposed to antibiotics in utero and 172 405 children (23.8%) were exposed during the first year of life. The risk of atopic dermatitis among children exposed to prenatal antibiotics was greater than that among children who were not exposed (adjusted hazard ratio [aHR], 1.10; 95% CI, 1.09-1.12). In the sibling-control analysis, no association was observed (aHR, 0.96; 95% CI; 0.92-1.00). Exposure to antibiotics during the first year of life was associated with a greater risk of atopic dermatitis (aHR, 1.52; 95% CI, 1.50-1.55), with attenuated associations in the sibling-control analysis (aHR, 1.24; 95% CI, 1.20-1.29).

**Conclusions and Relevance:**

In this cohort study, exposure to antibiotics in early life was associated with an increased risk of atopic dermatitis in the general Swedish population, but this risk was partially confounded by familial factors. Research on the ways in which antibiotic use and other shared familial factors affect other atopic diseases may be warranted.

## Key Points

**Question:**

Is there an association between exposure to antibiotics in utero and the first year of life with the risk of childhood atopic dermatitis?

**Findings:**

In this population-based cohort study of 722 767 singleton children in Sweden, exposure to antibiotics in utero and in the first year of life was associated with a modestly increased risk of atopic dermatitis. This risk decreased when siblings were compared, suggesting a shared familial liability.

**Meaning:**

The findings suggest that antibiotic use in prenatal and early postnatal life is associated with risk of atopic dermatitis in early childhood and that this association may be confounded in part by shared familial factors.

## Teaser

This cohort study examines the association between exposure to antibiotics in prenatal and early postnatal life and the development of atopic dermatitis during childhood.

## Introduction

Atopic dermatitis (eczema) is a common inflammatory skin disorder,^1,2^ with a global prevalence in children ranging from 7% to 25%, and is associated with significant morbidity and health care costs.^2,3^ Atopic dermatitis typically begins in early childhood and is characterized by fluctuating intensely pruritic skin lesions^4^ that may or may not persist into adulthood.^2^ Children who have atopic dermatitis with specific IgE antibodies are at an increased risk for development of allergic rhinitis, food allergies, and asthma.^4^

Atopic dermatitis has a multifactorial origin and is associated with genetic, inflammatory, and environmental factors.^2,5^ Although a familial (genetic) history of atopy is the main risk factor,^2^ several environmental factors are also associated with disease onset, but these associations are less consistent.^5^ One such factor is maternal use of antibiotics during pregnancy.^5,6,7^ Exposure to antibiotics in early life has been associated with delayed maturation of the gut microbiome, and the resulting disturbances may negatively affect bacterial diversity. These factors may be associated with altered intestinal compositional states in children and lead to atopy.^8^

Despite the potential disruption to the microbiome, prenatal exposure to antibiotics has not been consistently shown to be associated with atopic dermatitis and atopy.^2,6,9,10,11^ In a Belgian study of 773 children by Dom et al,^6^ prenatal exposure to antibiotics was found to be associated with atopic dermatitis (odds ratio [OR], 1.82; 95% CI, 1.14-2.92). However, conflicting results were found in other studies. A pooled analysis of 4 studies examining prenatal antibiotic use and atopic dermatitis conducted by Tsakok et al^9^ revealed no statistically significant association (OR, 1.30; 95% CI, 0.86-1.95), although the estimate was elevated. Similarly, Stensballe et al^12^ found that the prenatal use of antibiotics was not associated with risk of atopic dermatitis among children (hazard ratio [HR], 0.68; 95% CI, 0.43-1.09).

Postnatal exposure to antibiotics may also be associated with a risk of developing atopic dermatitis in early childhood. Some studies have shown that children who are treated with antibiotics in early childhood are at high risk of atopic dermatitis.^13,14,15,16^ In a study of 3306 participants by Mai et al^13^ and a study of 7916 Swedish twins and 35 365 Dutch twins by Slob et al,^14^ an increased risk was demonstrated. In the study by Mai et al,^13^ the adjusted HR (aHR) was 1.3 (95% CI, 1.1-1.5), and in the study by Slob et al,^14^ the adjusted OR (aOR) was 1.07 (95% CI, 1.01-1.14) for the Swedish twins and 1.08 (95% CI, 1.03-1.13) for the Dutch twins. However, in the study by Slob et al,^14^ after adjusting for shared familial factors in co-twins, no association was observed (Swedish twins: aOR, 1.76; 95% CI, 0.98-3.14; Dutch twins: aOR, 1.02; 95% CI, 0.69-1.52). Furthermore, the association could not be replicated by Kusel et al^17^ in a study of 198 participants (aOR, 1.02; 95% CI, 0.60-3.20) or by Kummeling et al^18^ in a study of 2764 participants (aOR, 0.94; 95% CI, 0.75-1.18). The inconsistent results observed in the different studies may be attributable in part to low statistical power in small studies, the use of different definitions of atopic dermatitis, restricted populations, and an inability to adjust for different familial and socioeconomic confounders.

To address the observed discrepancies, we conducted a large, population-wide, register-based cohort study of Swedish mother and child pairs to examine the association of antibiotic exposure in early life with the risk of atopic dermatitis in childhood. We further investigated this association within families using sibling control participants to adjust for familial factors and address potential associations.^19^

## Methods

### Study Population and Design

We performed a nationwide, prospective, register-based cohort study using both a general population birth cohort and sibling comparisons within the cohort. All Swedish singleton children born between March 1, 2006, and December 31, 2012 (n = 730 774), were included. Children were identified from the Medical Birth Register, which reports more than 98% of births in Sweden annually (established in 1973).^20^ Standardized maternal and child characteristics for the register are prospectively collected starting at the first prenatal care visit at the antenatal care clinic.^20^ Information about family relationships was retrieved from the Multi-Generation Register,^21^ which contains links between parent and child or children; vital statistics and migration information were retrieved from the Register of the Total Population.^22^ Institutional review board approval was obtained for this project from the Regional Ethics Review Board in Stockholm, Sweden. Informed consent was not required because the study did not involve human participants, and all data were deidentified before analyses were performed. This study followed the Strengthening the Reporting of Observational Studies in Epidemiology (STROBE) reporting guidelines.

The study period was selected to ensure full coverage of dispensed medications from the Swedish Prescribed Drug Register (established in July 2005)^23^ during the mothers’ pregnancy period and during early childhood. In addition, disease diagnoses from the antenatal period and early childhood were available from the National Patient Register, which has covered all inpatient diagnoses since 1987 and specialist outpatient care since 2001.^24^ This register does not include general practice outpatient care.

Children born abroad or whose parents immigrated during the pregnancy period were not included in the study to ensure availability of intrapartum events (n = 8006). All children were then followed up to emigration, death, or the study end on December 31, 2015 (n = 722 767) (eFigure 1 in the Supplement). We also identified 530 590 sibling combinations within the cohort, of which 74 663 were discordant. We linked the different national registers using the unique 10-digit personal identity number that is given to each Swedish resident at birth or immigration.

### Exposure

Maternal exposure to systemic antibiotics during pregnancy was defined as a dispensed prescription for antibacterial drugs. Antibiotics were identified using the Anatomic Therapeutic Chemical Classification System code J01. Using a classification developed by Marra et al^10^ and modified by Örtqvist et al,^25^ we classified the antibacterial drugs according to indication for use into 4 categories: airway, urinary tract, skin, and subcutaneous and other infections. In addition, we divided the aforementioned antibiotics into broad-spectrum and narrow-spectrum categories using a classification previously described by Örtqvist et al^25^ (eAppendix in the Supplement).

Start date of pregnancy was estimated as the date of birth minus the gestational age in days. Trimester in which antibiotic exposure occurred was determined by dividing the duration of pregnancy into 3 trimesters (first: 1-91 days; second: 92-189 days; third: ≥190 days).

The exposure of each child to systemic antibiotics during the first year of life was also investigated. An initial analysis examined the effects of use of any antibiotics during the first year of life. The antibacterial drugs were then classified according to indication for use, and the analysis was repeated.^10,25^ To account for the possible confounding effects of intrapartum exposure to antibiotics, we further adjusted for maternal use of antibiotics during pregnancy.

### Outcome

Atopic dermatitis was defined as a compound outcome extracted from the National Patient Register and the Swedish Prescribed Drug Register. According to an algorithm created by Henriksen et al,^26^ atopic dermatitis was defined using prespecified *International Statistical Classification of Diseases and Related Health Problems, Tenth Revision *codes and the Anatomic Therapeutic Chemical Classification System codes. The detailed algorithm and criteria are shown in the eAppendix in the Supplement. The first registered date of an atopic dermatitis diagnosis and/or the time to first dispensed prescription after baseline was considered the date of the outcome.

### Covariates

We selected covariates based on direct acyclic graphs to identify potential confounders recorded in the population registers (eFigure 2 in the Supplement). Independent variables extracted from the Medical Birth Register at baseline included the child’s sex (male or female), maternal age (continuous), family situation (mother living with father or other living situation), and parity (child’s birth order [continuous]). Maternal educational attainment (compulsory, ≤9 years; secondary, 10-11 years; or tertiary, ≥12 years) was extracted from the Longitudinal Integrated Database for Health Insurance and Labour Market Studies held by Statistics Sweden,^27^which was established in 1990. The area of residence at birth (Norrland, Götaland, or Svealand) was obtained from the Register of the Total Population. The other covariates, such as maternal smoking at first antenatal visit (yes or no), maternal history of asthma (yes or no), and mode of delivery (uncomplicated vaginal delivery, instrumental vaginal delivery, elective cesarean delivery, and emergency cesarean delivery) were derived from the Medical Birth Register.

### Statistical Analysis

We investigated the association of maternal use of antibiotics during pregnancy and antibiotic use during the first year of life with atopic dermatitis in the offspring separately. Cox proportional hazards regression with attained age as time scale was used to estimate HRs and 95% CIs for atopic dermatitis. The proportional hazards assumption was verified using log-log plots and Schoenfeld residuals plots. Participants were censored at emigration, death, or the end of the study (whichever came first).

For maternal antibiotic use during pregnancy, models were adjusted for sex and for multiple variables, including sex, birth weight, mother’s age, family situation, parity, level of education, area of residence, smoking history, maternal history of asthma, and mode of delivery. We included antibiotic use at any time during pregnancy and trimester-specific exposure as exposure variables. We then analyzed the association between maternal antibiotic use during pregnancy and atopic dermatitis by using the likely indication for antibiotic prescription as the exposure. For antibiotic use by the child during the first year of life, adjustments were made for similar covariates: sex, mother’s age, family situation, parity, level of education, area of residence, smoking history, maternal history of asthma, and mode of delivery. The models were additionally adjusted for maternal antibiotic use during pregnancy.

A sibling-control analysis was then conducted to account for a shared familial environment for both prenatal life and the first year of life as exposures. Sibling control participants were identified by shared maternity in the Multi-Generation Register. All sibling pairs were included in the analysis, but only those discordant for exposure and outcome were informative for the estimation of the HRs for exposure. The prenatal analysis models were adjusted for sex, mother’s age, family situation, smoking, and mode of delivery. In the models in which the first year of life was used as an exposure, maternal use of antibiotics was also included as a covariate. Analysis was done using a stratified Cox proportional hazards regression, and results were reported as HRs and 95% CIs.

We also included a sensitivity analysis that investigated the association of risk of atopic dermatitis with the number of courses of antibiotics dispensed during pregnancy. The association of use of broad-spectrum or narrow-spectrum antibiotics vs no antibiotic use with risk of atopic dermatitis was also assessed. Analyses were performed using Stata, version 16 (StataCorp LLC).^28^ Data were analyzed from June 1, 2020, to October 31, 2020.

## Results

Among the 722 767 singleton children included in the study, the mean (SD) age was 5.8 (2.4) years and 351 589 (48.6%) were female. During the follow-up period, 153 407 (21.2%) children were exposed to antibiotics in utero and 172 405 (23.8%) children were exposed to antibiotics during the first year of life. Mothers who used antibiotics were more likely to have a history of smoking than were those who did not (13 855 [9.0%] vs 37 405 [6.6%]) and were more likely to have a lower level of education (18 619 [12.1%] vs 55 410 [9.7%]). The exposed and unexposed groups were similar with regard to sex distribution, maternal age, maternal weight, and birth weight. The baseline characteristics of the study population are shown in Table 1.

**Table 1.** Baseline Characteristics of the Study Population by Antibiotic Exposure During Pregnancy

Characteristic	Children or mothers^a^	
	No antibiotic use (n = 569 360)	Antibiotic use (n = 153 407)
Child’s age, median (SD), y	5.8 (2.4)	5.9 (2.5)
Sex of child		
Male	290 344 (51.0)	80 834 (52.7)
Female	279 016 (49.0)	72 573 (47.3)
Birth weight, median (SD), g^b^	3540 (578)	3540 (585)
Mother’s age, median (SD), y	30 (5.2)	30 (5.4)
Mother’s BMI, median (SD)^c^	23.6 (4.6)	23.8 (4.9)
Family situation^d^		
Mother living with child’s father	512 937 (90.1)	135 535 (88.4)
Other	31 581 (5.6)	10 736 (7.0)
Parity		
1	258 432 (45.4)	63 164 (41.2)
2	206 729 (36.3)	58 202 (37.9)
3	75 340 (12.9)	22 117 (14.4)
≥4	30 859 (5.4)	9924 (6.5)
Maternal educational level^e^		
Primary	55 410 (9.7)	18 619 (12.1)
Secondary	210 558 (37.0)	59 663 (38.9)
Tertiary or higher	296 923 (52.2)	73 522 (47.9)
Area of residence		
Norrland	62 480 (11.0)	15 164 (9.9)
Svealand	235 397 (41.3)	66 617 (43.4)
Götaland	271 404 (47.7)	71 606 (46.7)
Exposed to smoking^f^	37 405 (6.6)	13 855 (9.0)
Maternal asthma	33 826 (5.9)	13 485 (8.8)
Mode of delivery^g^		
Uncomplicated vaginal	429 567 (75.4)	113 270 (73.8)
Instrumental vaginal	43 939 (7.7)	11 299 (7.4)
Emergency cesarean delivery	55 818 (9.8)	15 874 (10.4)
Elective cesarean delivery	39 755 (7.0)	12 862 (8.4)
Atopic dermatitis	63 133 (11.1)	19 141 (12.5)
Children who died during follow-up	1590 (0.3)	458 (0.3)

Abbreviation: BMI, body mass index (calculated as weight in kilograms divided by height in meters squared).

^a^ Data are presented as number (percentage) of children or mothers unless otherwise indicated.

^b^ Data missing for 1078 children.

^c^ Data missing for 57 618 mothers.

^d^ Data missing for 31 978 children.

^e^ Data missing for 8072 mothers.

^f^ Data missing for 27 641 children.

^g^ Data missing for 383 mothers.

During 4 175 474 person-years of follow-up, antibiotic use at any time during pregnancy was associated with a 12% higher rate of atopic dermatitis in crude analyses (aHR, 1.12; 95% CI, 1.11-1.14) and a 10% higher rate in multivariable-adjusted analyses (aHR, 1.10; 95% CI, 1.09-1.12) (Table 2). When the trimester of antibiotic use was used as an exposure, the risk of atopic dermatitis was increased in the multivariable-adjusted models regardless of trimester of use (first trimester: aHR, 1.10 [95% CI, 1.07-1.13]; second trimester: aHR, 1.08 [95% CI, 1.06-1.11]; and third trimester: aHR, 1.12 [95% CI, 1.09-1.14]). Adjusting for mode of delivery also did not change the point estimates (Table 2).

**Table 2.** Antibiotic Exposure in Different Stages of Pregnancy and the Risk of Atopic Dermatitis

Antibiotic exposure	Children, No. (%)		Hazard ratio (95% CI)		
	Exposed to antibiotics	Exposed to antibiotics and developed atopic dermatitis	Age-adjusted	Adjusted^a^	Adjusted^b^
Any time during pregnancy	153 407 (21.2)	19 141 (12.5)	1.12 (1.11-1.14)	1.11 (1.09-1.13)	1.10 (1.09-1.12)
First trimester	58 003 (37.8)	7314 (12.5)	1.12 (1.10-1.15)	1.10 (1.08-1.13)	1.10 (1.07-1.13)
Second trimester	67 519 (44.0)	8467 (12.5)	1.11 (1.09-1.14)	1.09 (1.06-1.11)	1.08 (1.06-1.11)
Third trimester	62 146 (40.5)	7984 (12.8)	1.14 (1.12-1.17)	1.12 (1.09-1.15)	1.12 (1.09-1.14)

^a^ Adjusted for age, sex, mother’s age, family situation, parity, level of education, area of residence, smoking history, and maternal history of asthma.

^b^ Adjusted for age, sex, mother’s age, family situation, parity, level of education, area of residence, smoking history, maternal history of asthma, and mode of delivery.

In analyses using the likely infection for which maternal antibiotics were prescribed as the exposure, an increased risk of atopic dermatitis was observed across all categories: respiratory infections (aHR, 1.08; 95% CI, 1.06-1.11), urinary tract infections (aHR, 1.15; 95% CI, 1.11-1.19), skin or soft-tissue infections (aHR, 1.12; 95% CI, 1.06-1.19), or other conditions (aHR, 1.19; 95% CI, 1.12-1.26) (eTable 1 in the Supplement). In addition, increased number of antibiotic courses prescribed to the mother during pregnancy was associated with greater risk of atopic dermatitis in the offspring (1-2 courses: aHR, 1.10 [95% CI, 1.08-1.12]; 3-4 courses:a HR, 1.12 [95% CI, 1.07-1.18]; ≥5 courses: aHR, 1.24 [95% CI, 1.15-1.35]) (eTable 2 in the Supplement).

In the sibling-control analysis, no association between antibiotic use during pregnancy and risk of atopic dermatitis was observed. This observation remained regardless of the stage of pregnancy (age-adjusted: HR, 0.97 [95% CI, 0.94-1.00]; multivariable analyses: aHR, 0.96 [95% CI, 0.92-1.00]) (Table 3).

**Table 3.** Sibling Analysis of the Association Between Antibiotic Exposure in Different Stages of Pregnancy and Risk of Atopic Dermatitis

Antibiotic exposure	Discordant siblings exposed to antibiotics, No. (%)	Hazard ratio (95% CI)		
		Age-adjusted	Adjusted^a^	Adjusted^b^
Any time during pregnancy	10 270 (13.8)	0.97 (0.94-1.00)	0.96 (0.92-0.99)	0.96 (0.92-1.00)
First trimester	4002 (39.0)	0.96 (0.91-1.01)	0.94 (0.90-1.00)	0.95 (0.91-1.01)
Second trimester	4573 (44.5)	0.96 (0.92-1.01)	0.96 (0.91-1.01)	0.96 (0.91-1.01)
Third trimester	4366 (42.5)	0.97 (0.93-1.02)	0.95 (0.90-1.00)	0.95 (0.90-1.00)

^a^ Adjusted for age, sex, mother’s age, family situation, parity, and smoking history.

^b^ Adjusted for age, sex, mother’s age, family situation, parity, smoking history, and mode of delivery.

During 4 175 474 person-years of follow-up, the use of antibiotics during the first year of life was associated with an increased risk of atopic dermatitis in multivariable-adjusted analyses (HR, 1.52; 95% CI, 1.50-1.55). In multivariable-adjusted analyses in which the type of infection for which antibiotics were prescribed during the first year of life was used as the exposure, an increased risk of atopic dermatitis was observed across all categories: respiratory (aHR, 1.45; 95% CI, 1.43-1.47), urinary tract (aHR, 1.33; 95% CI, 1.25-1.42), and skin or soft-tissue (aHR, 2.93; 95% CI, 2.81-3.06) infections (eTable 3 in the Supplement). For children who received antibiotics during the first year of life, the sibling-control analysis showed that the increased risk of atopic dermatitis remained, although it was attenuated in multivariable-adjusted analyses (aHR, 1.24; 95% CI, 1.20-1.29).

Sensitivity analysis showed that when children exposed to antibiotics were compared with children who had not been exposed to antibiotics in utero, no difference in risk between children exposed to broad-spectrum antibiotics (HR, 1.08; 95% CI, 1.04-1.14) and those exposed to narrow-spectrum antibiotics (HR, 1.08; 95% CI, 1.06-1.10) was observed; however, the risk of atopic dermatitis was higher among children exposed to broad-spectrum or narrow-spectrum antibiotics during the first year of life compared with children not exposed to any antibiotics during the first year of life even after adjustment for maternal use of antibiotics during pregnancy (broad-spectrum vs no antibiotics: aHR, 1.60 [95% CI, 1.57-1.63]; narrow-spectrum vs no antibiotics: aHR, 1.41 [95% CI, 1.38-1.45]) (eTable 4 in the Supplement). When adjusting the sibling relationship, no association was observed with antibiotic use during pregnancy; however, among children exposed to antibiotics during the first year of life compared with children not exposed to antibiotics, the risk of atopic dermatitis among children exposed to broad-spectrum antibiotics was similar to that among children exposed to narrow-spectrum antibiotics (eTable 5 in the Supplement).

## Discussion

In this large, population-based cohort study, we found that maternal use of antibiotics during pregnancy was associated with a higher risk of atopic dermatitis during early childhood. There was a dose-dependent association of maternal antibiotic use with risk of atopic dermatitis in childhood. The positive association observed in the whole-cohort analyses with multivariable adjustment did not persist in the sibling- control analyses, in which the risks were significantly attenuated and, in some cases, reduced to null. We also found that use of antibiotics during the first year of life was associated with an increased risk of atopic dermatitis in children, and this association persisted even after controlling for familial and environmental factors in the sibling-control analysis, although the magnitude was attenuated.

### Comparison With Other Studies on Antibiotic Use In Utero

Studies conducted on prenatal antibiotic exposure and the risk of atopic dermatitis have had inconsistent results.^6,12,29^ In a study of approximately 31 000 Danish children, Stensballe et al^12^ found no association between maternal use of antibiotics during the third trimester and risk of atopic dermatitis during childhood. In addition to the factors that we adjusted for, they adjusted for diet, daycare attendance, and seasonality; however, their study population was smaller and had a shorter follow-up period. In contrast, in a study by Timm et al^30^ of 62 560 Danish children and another by Dom et al^6^ of 773 children from a Belgian prospective birth cohort,^6^ the investigators determined that children exposed to antibiotics in utero had a high risk of atopic dermatitis. In the study by Timm et al,^30^ only children born to mothers with atopy who had used antibiotics in all 3 trimesters were at increased risk for atopic dermatitis. This finding suggests a possible dose-response relationship and also that maternal allergy may be associated with increased effects of environmental exposures in the offspring.^30^ Although we adjusted for maternal history of asthma and did not adjust for maternal atopy, we still found an increased risk of eczema in children whose mothers had more prescriptions.

In addition, similar to the present study, children in the study by Dom et al^6^ were followed up from birth to at least 4 years, and these investigators also corrected for postnatal exposure to antibiotics. Our study replicated these results, and adjustment for the trimester of exposure, the likely indication for antibiotic use, and the use of broad- or narrow-spectrum antibiotics did not alter our findings of greater risk. Prenatal exposure to antibiotics may modify gut microbial diversity and delay its maturation.^8^ This alteration may be associated with immune dysregulation that leads to atopic dermatitis and other atopic conditions in offspring.^31^

Although some previous studies adjusted for birth order or bacterial infections in siblings, to our knowledge, no sibling analysis or co-twin studies investigating prenatal antibiotic use and the risk of atopic dermatitis have been published. The lack of association observed in our study shows that familial and environmental confounding may have affected our observations.

### Comparison With Other Studies on Antibiotics Use in the First Year of Life

In a study by McKeever et al^7^ of 29 238 British children, no association between early life exposure to antibiotics and atopic dermatitis was demonstrated. The longitudinal study included adjustment for birth order effects and antibiotic dose-response relationships; however, their results were likely confounded by parental hospital consulting behavior and thus possible underreporting.^7^ Conversely and similar to our study findings, in a study by Metzler et al^32^ of 1080 unrelated children from a European birth cohort, treatment with antibiotics in the first year of life was associated with increased risk of atopic dermatitis at different time points up to 4 years of age even after exclusion of children with atopic dermatitis in the first year of life.

Similar to our results, a study by Slob et al^14^ in 7916 discordant Swedish twins showed that antibiotic use in the first years of life was associated with an increased risk of atopic dermatitis even after controlling for genetic and environmental confounders. In addition, they found a greater risk of atopic dermatitis in their co-twin analyses,^14^ which was also observed in our sibling-control analyses. Twin pairs are known to have more concurrently shared familial and genetic environments than non-twin siblings. The possibility of unmeasured confounders that change across pregnancies and across circumstances may be associated with upbringing cannot be ruled out in non-twin siblings.^33^

### Strengths and Limitations

This study has strengths. The large, register-based population allowed for a long follow-up period while minimizing both selection and recall bias. The registers provided access to a large number of confounding factors and allowed us to explore the role of familial factors. The sibling-control design allowed us to adjust for unmeasured genetic and environmental confounders shared by siblings.^19^ We were able to adjust for some important covariates, including maternal history of asthma, mode of delivery, maternal history of tobacco use, and area of residence. In an additional analysis, we adjusted for level of education, but this did not alter the main results.

This study also has limitations. We were not able to adjust for all variables; important variables, such as cigarette smoking, which is known to be associated with atopic dermatitis, were only available at antenatal booking, and no information was available about exposure during the first year of life.^34^ However, by performing the sibling analysis, we tried to account for smoking during the first year of life for both siblings. In addition, we could not rule out the possibility of residual confounding owing to factors not shared by siblings. We are aware that use of the sibling-control design may have resulted in some potential crossover effects and that the analysis design is more sensitive to measurement error.^35^ However, we are confident that the use of sibling comparisons accounted for some important genetic variants and shared environments. Although well-established, validated definitions for diagnosis of established atopic dermatitis exist, finding validated measures of eczema at the population level for children remains a challenge. In this study, we used the algorithm created by Henriksen et al.^26^ They included more than 1.5 million children in their cross-national, register-based cohort study, with approximately 530 000 of those children born in Sweden and overlapping with the population in the present study. We used longer follow-up period, a larger Swedish-born population, and an added sibling analysis.

Another important limitation was potential misclassification of the outcome. Atopic dermatitis medication is usually supplied over the counter, and in general, parents do not take their children to a health care facility for minor cases of the condition. Thus, it is possible that only pediatric patients with the most severe forms of atopic dermatitis or those seen for other medical conditions are reported in the Prescribed Drug Register and National Patient Register. It is also possible that the exposure of 1 sibling influenced the outcome of another.^19^

## Conclusions

In this cohort study, antibiotic use in prenatal and early postnatal life was associated with risk of atopic dermatitis in early childhood. Although the association between maternal antibiotic use at any time during pregnancy and atopic dermatitis during childhood was confounded by familial (genetic and environmental) factors, the use of antibiotics during the first year of life was associated with risk of atopic dermatitis, although it was partly confounded by familial factors. Further research should be conducted to determine the underlying mechanisms and the atopic dermatitis phenotypes that are more likely to be associated with other conditions, such as asthma and allergic rhinitis.

## Notes

Supplement. **eAppendix.** Henriksen Criteria **eTable 1.** Maternal Antibiotics Use by Indication **eTable 2.** Number of Courses of Antibiotics at Any Time in Pregnancy **eTable 3.** Childhood Antibiotics Use by Indication at Any Point During the First Year of Life **eTable 4.** Exposure to Narrow and Broad Spectrum Antibiotics in Utero and During the First Year of Life and the Risk of Atopic Dermatitis in Early Childhood **eTable 5.** Sibling Analysis Examining Maternal Antibiotics Use and Antibiotics Use During the First Year of Life and the Risk of Atopic Dermatitis **eFigure 1.** Flow Chart Showing Eligible Population **eFigure 2.** Direct Acyclic Graph **eReferences.**
