# Supplementary material for: Association of Early Life Exposure to Antibiotics With Risk of Atopic Dermatitis in Sweden
Source: JAMA Netw Open. 2021 Apr 29;4(4):e215245. doi: 10.1001/jamanetworkopen.2021.5245 (PMC8085722; doi:10.1001/jamanetworkopen.2021.5245)
Supplement: Supplement. — eAppendix. Henriksen Criteria eTable 1. Maternal Antibiotics Use by Indication eTable 2. Number of Courses of Antibiotics at Any Time in Pregnancy eTable 3. Childhood Antibiotics Use by Indication at Any Point During the First Year of Life eTable 4. Exposure to Narrow and Broad Spectrum Antibiotics in Utero and During the First Year of Life and the Risk of Atopic Dermatitis in Early Childhood eTable 5. Sibling Analysis Examining Maternal Antibiotics Use and Antibiotics Use During the First Year of Life and the Risk of Atopic Dermatitis eFigure 1. Flow Chart Showing Eligible Population eFigure 2. Direct Acyclic Graph eReferences. [file jamanetwopen-e215245-s001.pdf]

Supplementary Online Content

Mubanga M, Lundholm C, D'Onofrio BM, Stratmann M, Hedman A, Almqvist C. Association of early life exposure to antibiotics with risk of atopic dermatitis in Sweden. *JAMA Netw Open*. 2021;4(4):e215245. doi:10.1001/jamanetworkopen.2021.5245

eAppendix. Henriksen Criteria

eTable 1. Maternal Antibiotics Use by Indication

eTable 2. Number of Courses of Antibiotics at Any Time in Pregnancy

eTable 3. Childhood Antibiotics Use by Indication at Any Point During the First Year of Life

eTable 4. Exposure to Narrow and Broad Spectrum Antibiotics in Utero and During the First Year of Life and the Risk of Atopic Dermatitis in Early Childhood

eTable 5. Sibling Analysis Examining Maternal Antibiotics Use and Antibiotics Use During the First Year of Life and the Risk of Atopic Dermatitis

eFigure 1. Flow Chart Showing Eligible Population

eFigure 2. Direct Acyclic Graph

eReferences.

This supplementary material has been provided by the authors to give readers additional information about their work.

© 2021 Mubanga M et al. *JAMA Network Open*.

eAppendix.

Henriksen Criteria[1]

We used the Henriksen criteria to identify children with atopic dermatitis.[1] The algorithm for this criteria is based on specific ICD-10 codes from National Patient Register and ATC codes from the Swedish Prescribed Drug Register. A diagnosis of atopic dermatitis was generated in three steps (1) the selection of disease-specific hospital diagnoses and disease specific medication; (2) application of criteria of repetition of the disease-specific medication within 12 months because of chronicity and recurrence, and (3) exclusion of other medical conditions known to lead to use of the medication types used as inclusion criteria in step 1.

Criteria 1 (ICD-10)
≥1 hospital contact for: atopic dermatitis (L20) or winter feet (L308).

Criteria 2 (based on ATC codes)
≥1 filled prescription of: agents for dermatitis: tacrolimus, pimecrolimus (D11AH) without any of the exclusion criteria as specified below

Criteria 3 (based on ATC codes)
≥ 2 filled prescriptions of: corticosteroids for topical use (D07) within 12 months without any of the exclusion criteria as specified below.

Without co-occurring hospital contacts and/ or combination of filled prescriptions below (exclusions criteria):
Children ***without*** atopic dermatitis (L20) with a diagnosis of:
Seborrhoeic dermatitis (L21), diaper dermatitis (L22), allergic contact dermatitis (L23), irritant contact dermatitis (L24), unspecified contact dermatitis (L25), exfoliative dermatitis (L26), dermatitis due to substances taken internally (L27), lichen simplex chronicus and prurigo (L28), pruritus (L29), other dermatitis (L30 except L308C), papulosquamous disorders (L40 – L45), other erythematosus conditions (L53), sunburn (L55), other acute skin changes due to ultraviolet radiation (L56), vitiligo (L80), atrophic disorders of the skin (L90) and lupus erythematosus (L93).
These may or may not be combined with the following criteria:

Exclusion medication criteria*:*
≥1 filled prescription of either:
Anti-psoriasics (D05) or salicylates for dermatological use (D02AF) or corticosteroids moderate or potent other combinations (D07XB) or corticosteroids moderate or potent other combinations (D07XC) or corticosteroids (group IV) clobetasol (D07AD01) or clobetasol and antibiotics (D07CD01) ***AND*** antifungals (D01) (implies corticosteroid (group IV) use for vaginal fungal infection).

*For prescription of corticosteroid group IV, a filled prescription of group I-III should also be used (as atopic dermatitis is never treated alone with group IV)

Systemic Antibacterial Drugs Used

Based on the classification by Marra[2] et al and modified by Örtqvist et al[3], we classified the antibiotics used in the study into 4 categories (1) *drugs used in common airway infection* (penicillin, amoxicillin, cephalosporin, and macrolides), (2) *drugs used to treat urinary tract infections (*sulphonamides, Pivmecillinam, trimethoprim , ciprofloxacin, norfloxacin and nitrofurantoin), (3) *drugs used in treating skin and soft tissue infections* **(**dicloxacillin, cloxacillin, and flucloxacillin) (4) other infections (antibiotics not included in the above classifications). Furthermore, we divided the aforementioned antibiotics into broad and narrow spectrum categories using a classification previously described for use by Örtqvist et al.[3] Narrow spectrum antibiotics included (pivmecillinam, penicillin, dicloxacillin, cloxacillin, flucloxacillin, cephalexin, cefadroxil, erythromycin, roxitromycin,

© 2021 Mubanga M et al. *JAMA Network Open*.

clarithromycin, azithromycin clindamycin, vancomycin, teicoplanin, fusidinsyra, nitrofurantoin) and broad spectrum antibiotics included (doxycycline, lymecyclin, oxitetracycline, tetracycline, pivampicillin, amoxicillin,amimox and beta lactamase, piperacillin and beta lactamase inhibitor, cefuroxime, loracabef, cefotaxime, ceftazidime, ceftriaxone, cefpodoxime, ceftibuten, ceftriaxone, meronem, ertapenem, impenem beta lactamase inhibitor trimethoprim, sulfonamide plus trimethoprim, tobramycin, gentamycin, amikacin, ofloxacin, ciprofloxacin, norfloxacin, levofloxacin, moxifloxacin and kolistin).

eTable1. Maternal antibiotics use by indication at any point during pregnancy and the risk of atopic dermatitis

Antibiotics Exposure	Exposed to antibiotics (n)	Age-Adjusted* HR (95% CI)	Adjusted† HR (95% CI)	Adjusted‡ HR (95% CI)
No antibiotics	569,360	**Ref**	**Ref**	**Ref**
Airway disease	107,513	1.09 (1.07 – 1.12)	1.09 (1.07 – 1.11)	1.08 (1.06 – 1.11)
Urinary Tract Infections	27,335	1.18 (1.15 – 1.23)	1.16 (1.12 – 1.20)	1.15 (1.11 – 1.19)
Skin or soft tissue inf.	9,867	1.18 (1.12 – 1.25)	1.13 (1.07 -1.20)	1.12 (1.06 – 1.19)
Other infections	8,692	1.27 (1.20 -1.34)	1.20 (1.13 – 1.27)	1.19 (1.12 – 1.26)

*Adjusted for age
†Adjusted for age, sex, mother's age, family situation, parity, level of education, area of residence, smoking history and maternal history of asthma.
‡ Adjusted for age, sex, mother's age, family situation, parity, level of education, area of residence, smoking history, maternal history of asthma and mode of delivery

eTable2. Adjusted risks associated with the number of courses of antibiotics at any time in pregnancy.

Antibiotics Exposure	Total Population exposed	Age-Adjusted* HR (95% CI)	Adjusted† HR (95% CI)
**Number of Antibiotic prescriptions**			
None	569,360 (78.8)	Ref	Ref
1-2	134,848 (18.7)	1.11 (1.09 – 1.13)	1.10 (1.08 – 1.12)
3-4	14,054 (1.9)	1.18 (1.13 – 1.24)	1.12 (1.07 – 1.18)
>5	4,505 (0.6)	1.34 (1.24 – 1.45)	1.24 (1.15 – 1.36)

*Adjusted for age
†Adjusted for age, sex, mother's age, family situation, parity, level of education, area of residence, smoking history, maternal history of asthma and mode of delivery

eTable3. Childhood antibiotics use by indication at any point during the first year of life and the risk of atopic dermatitis

Antibiotics Exposure	Exposed to antibiotics (n)	Age-Adjusted* HR (95% CI)	Adjusted† HR (95% CI)	Adjusted‡ HR (95% CI)
No antibiotics	550,371 (76.1)	**Ref**	**Ref**	**Ref**
Airway disease	155,336 (21.5)	1.46 (1.43 – 1.48)	1.45 (1.43 – 1.48)	1.45 (1.43 – 1.47)
Urinary Tract Infections	7,710 (1.1)	1.34 (1.26 – 1.43)	1.34 (1.26 – 1.43)	1.33 (1.25 – 1.42)
Skin or soft tissue inf.	9,350 (1.3)	2.96 (2.84 – 3.09)	2.95 (2.81 – 3.07)	2.93 (2.81 – 3.06)

*Adjusted for age
†Adjusted for age and maternal use of antibiotics
‡ Adjusted for age, maternal use of antibiotics, sex, mother's age, family situation, parity, level of education, area of residence, smoking history, maternal history of asthma and mode of delivery

© 2021 Mubanga M et al. *JAMA Network Open*.

eTable4. Exposure to narrow and broad spectrum antibiotics in utero and during the first year of life and the risk of atopic dermatitis in early childhood

Antibiotics Exposure	Total Population exposed	Age-Adjusted* HR (95% CI)	Adjusted† HR (95% CI)	††Adjusted for maternal exposure during pregnancy
**Maternal exposure§ during pregnancy**				
No antibiotics	569,356 (78.8)	REF	REF	REF
Narrow spectrum	95,988 (13.3)	1.08 (1.06-1.11)	1.08 (1.06-1.14)	-
Broad spectrum	22,097 (3.0)	1.12 (1.08-1.17)	1.08 (1.04-1.12)	-
**Exposure during first year of life**				
No antibiotics	550,360 (76.2)	REF	REF	REF
Narrow spectrum	76,276 (10.6)	1.40 (1.39-1.43)	1.41 (1.38-1.44)	1.41 (1.38-1.45)
Broad spectrum	96,120 (13.3)	1.62 (1.59-1.65)	1.61 (1.58-1.64)	1.60 (1.57-1.63)

***Adjusted for age**
†Adjusted for age, sex, mother's age, family situation, parity, level of education, area of residence, smoking history, maternal history of asthma and mode of delivery
†† Adjusted for age, sex, mother's age, family situation, parity, level of education, area of residence, smoking history, maternal history of asthma, mode of delivery and maternal use of antibiotics in pregnancy.
§35,326 undetermined spectrum information

eTable5. Sibling analysis examining maternal antibiotics use and antibiotics use during the first year of life and the risk of atopic dermatitis in early childhood.

Antibiotics Exposure	Total Population exposed	Age-Adjusted* HR (95% CI)	Adjusted† HR (95% CI)	††Adjusted for maternal exposure during pregnancy
**Maternal exposure during pregnancy**				
No antibiotics	308,993 (84.6)	REF	REF	-
Narrow spectrum	45,418 (12.4)	0.97 (0.93-1.02)	0.97 (0.93-1.01)	-
Broad spectrum	10,650 (2.9)	0.99 (0.92-1.08)	0.97 (0.89-1.06)	-
**Exposure during first year of life**				
No antibiotics	276,656 (75.2)	REF	REF	REF
Narrow spectrum	41,518 (11.3)	1.21 (1.16-1.26)	1.28 (1.22-1.34)	1.28 (1.22-1.34)
Broad spectrum	49,857 (13.5)	1.22 (1.17-1.27)	1.21 (1.16-1.27)	1.21 (1.16-1.27)

*Adjusted for age
†Adjusted for age, sex, mother's age, family situation and mode of delivery
††Adjusted for age, sex, mother's age, family situation, smoking, mode of delivery and maternal use of antibiotics in pregnancy.

© 2021 Mubanga M et al. *JAMA Network Open*.

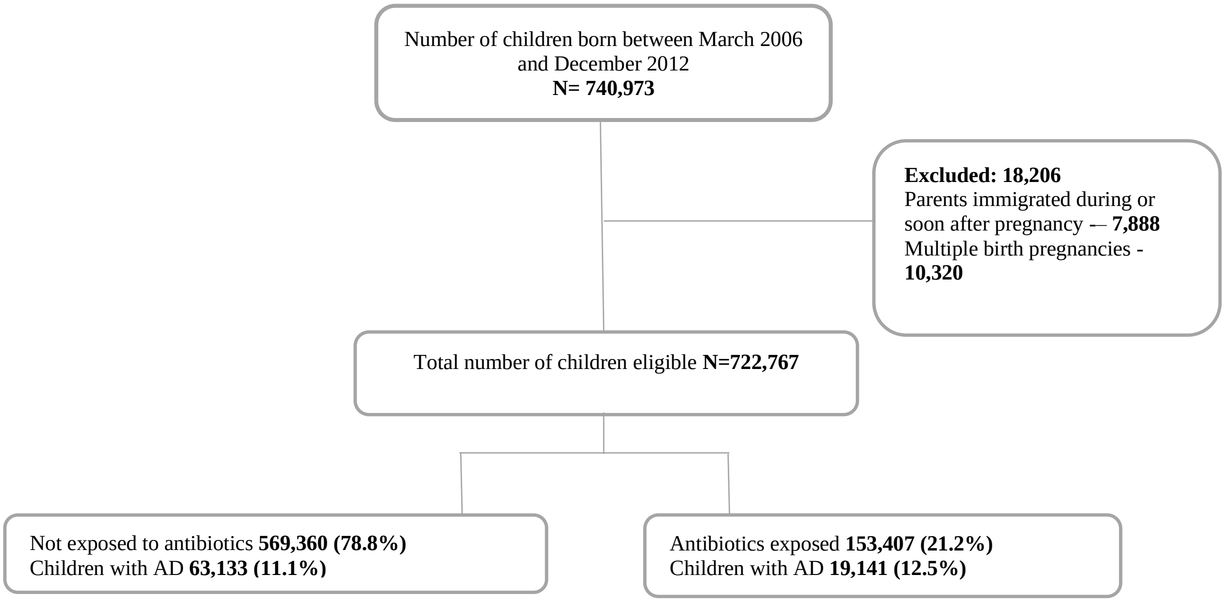

eFigure1. Flow Chart showing eligible population.

© 2021 Mubanga M et al. *JAMA Network Open*.

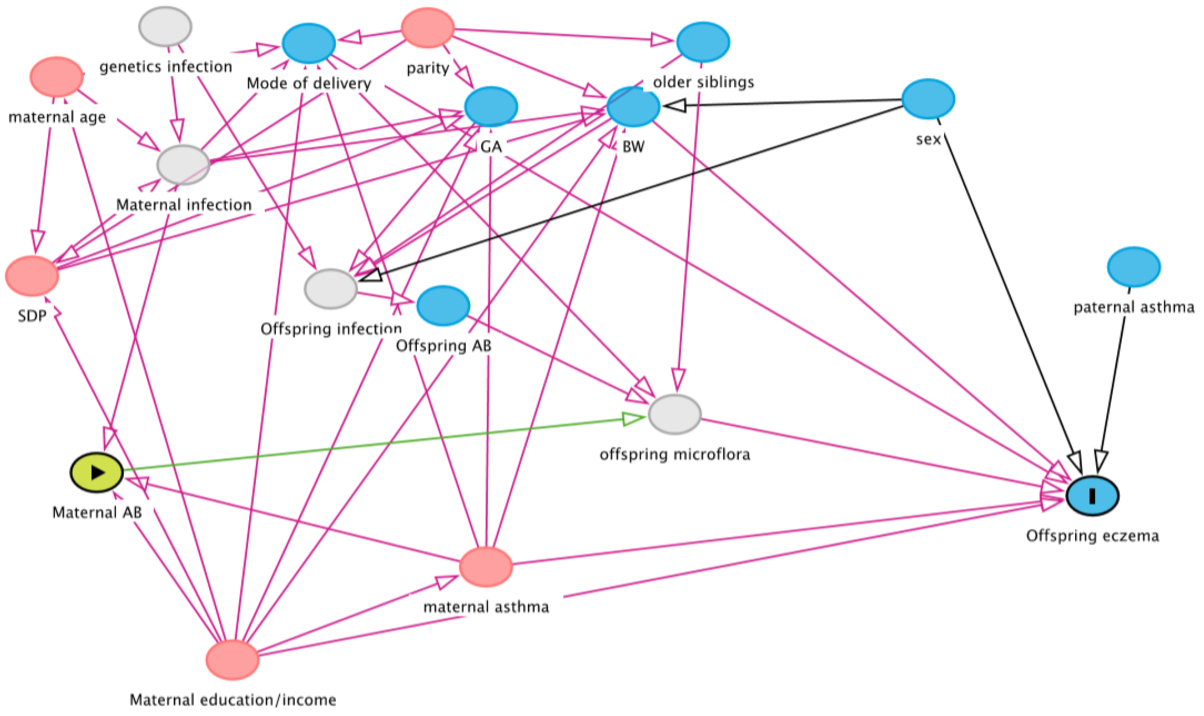

eFigure2: Direct Acyclic Graph

eReferences

1. Henriksen L, Simonsen J, Haerskjold A, et al. Incidence rates of atopic dermatitis, asthma, and allergic rhinoconjunctivitis in Danish and Swedish children. *Journal of Allergy and Clinical Immunology.* 2015;136(2):360-366.e362.
2. Marra F, Marra CA, Richardson K, et al. Antibiotic use in children is associated with increased risk of asthma. *Pediatrics.* 2009;123(3):1003-1010.
3. Örtqvist AK, Lundholm C, Kieler H, et al. Antibiotics in fetal and early life and subsequent childhood asthma: nationwide population based study with sibling analysis. *BMJ : British Medical Journal.* 2014;349:g6979.

© 2021 Mubanga M et al. *JAMA Network Open*.
